# *QuickStats:* Age-Adjusted Percentage* of Adults Aged ≥25 Years Who Were Told in the Past 12 Months by a Doctor or Other Health Professional That They Had a Liver Condition,^†^ by Education Level — National Health Interview Survey,^§^ 2016

**DOI:** 10.15585/mmwr.mm6713a6

**Published:** 2018-04-06

**Figure:**
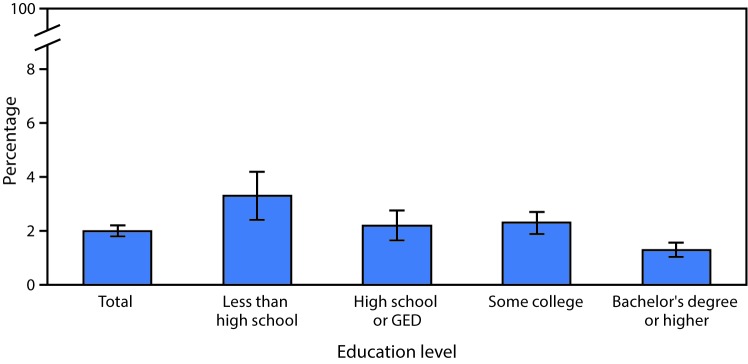
In 2016, 2.0% of adults aged ≥25 years who were surveyed had been told by a doctor or other health professional in the past 12 months that they had a liver condition. The prevalence of liver condition declined as education level increased. Adults who had completed a bachelor’s degree or higher were the least likely to have been diagnosed with any liver condition (1.3%), whereas those without a high school diploma were the most likely (3.3%).

**Source:** Tables of summary health statistics for U.S. adults, National Health Interview Survey, 2016. https://www.cdc.gov/nchs/nhis/SHS/tables.htm.

